# The Effect of Waterpipe Tobacco Smoking on Bone Healing Following Femoral Fractures in Male Rats

**DOI:** 10.3389/fsurg.2021.722446

**Published:** 2021-10-04

**Authors:** Amirreza Sadeghifar, Mohamad Sheibani, Siyavash Joukar, Shahriar Dabiri, Samanehsadat Alavi, Omid Azari, Darioush Vosoghi, Yas Zeynali, Yasman Zeynali, Mohamad Shahraki, Amirhesam Torghabe, Farzaneh Rostamzadeh, Alireza Nasri

**Affiliations:** ^1^Orthopedic Department, Afzalipour School of Medicine, Kerman University of Medical Sciences, Kerman, Iran; ^2^Physiology Research Center, Institute of Basic and Clinical Physiology Sciences, and Department of Physiology and Pharmacology, Afzalipour School of Medicine, Kerman University of Medical Sciences, Kerman, Iran; ^3^Pathology Department and Stem Cell Research Center, Afzalipour School of Medicine, Kerman University of Medical Sciences, Kerman, Iran; ^4^Cardiovascular Research Center, Institute of Basic and Clinical Physiology Sciences, Kerman University of Medical Sciences, Kerman, Iran; ^5^Department of Clinical Sciences, Faculty of Veterinary Medicine, Shahid Bahonar University of Kerman, Kerman, Iran

**Keywords:** waterpipe tobacco smoking, femur fracture, union, angiogenesis markers, insulin growth factor-1, TGF-β

## Abstract

**Background:** Given the increasing use of waterpipe tobacco smoking in the world and its unknown effects on bone healing, this study investigated the repairing of femoral bone fractures in rats exposed to waterpipe tobacco smoking (WTS).

**Main Methods:** This study involved 40 male Wistar rats that were divided into two groups, including the femoral fracture (Fx) and the Fx + WTS groups. Each group was divided into two subgroups that were evaluated for bone healing 28 and 42 days after femoral fracture. After fixing the fractured femur, the healing process was evaluated by radiography, pathological indicators, and a measurement of the blood levels of vascular endothelial growth factor (VEGF), parathyroid hormone (PTH), Ca ++, transforming growth factor-beta (TGF-β), and insulin-like growth factor 1 (IGF-1). Additionally, the density of VEGF and CD34 in fracture tissue was investigated by immunohistochemistry.

**Key Findings:** Radiographic findings showed that factors related to the earlier stages of bone healing had higher scores in the Fx + WTS28 and 42 subgroups in comparison to the Fx groups. The density of VEGF and CD34 showed that the angiogenesis processes were different in the bone fracture area and callus tissue in the Fx +WTS subgroups. The serum levels of VEGF, TGF-β, and IGF-1 were significantly lower in the Fx +WTS42 group, and PTH in the Fx +WTS28 group was higher than that in the other groups.

**Significance:** The findings showed the disturbance and delay in the femoral fracture union in rats exposed to hookah smoke. This is partly due to the reduction of molecular stimuli of bone synthesis and the attenuation of quantitative angiogenesis.

## Introduction

Tobacco consumption is one of the most important effective factors in causing disease, especially cardiovascular and pulmonary diseases, disability, and premature death (1). Although cigarette smoking is more common among smokers, hookah use is on the rise in many parts of the world, especially among young people. According to popular belief, the harmful effects of waterpipe tobacco smoking (WTS) are less than cigarettes because tobacco smoke passes through the water tank and its harmful substances are purified (1, 2). On the contrary, studies show that smoking tobacco in the form of hookah can have more toxic effects and harms. Hookah users are about 56 times more exposed to smoke and 4 times more to carbon monoxide (CO) than cigarette smokers (2).

Cigarette smoking increases the risk of osteoporosis and bone fractures due to changes in the biological functions of the whole body or factors that regulate bone metabolism and bone turnover (3). In addition, clinical studies and animal models indicate that cigarette smoke and nicotine delay the healing of bone fractures (3, 4). A literature review reported that patients with open and high-grade fractures need more time to heal if they smoke because they have higher levels of catecholamines, less tissue blood flow, and are more prone to bone infections (3). Additionally, a study showed that nicotine increases angiogenesis but reduces the expression of bone morphogenetic protein 2 and impairs bone healing (4).

Bone regeneration and fracture healing are affected by growth factors and cytokines (5). Vascular endothelial growth factor (VEGF), one of the most important factors in vascular growth, decreases in the presence of nicotine (6, 7). Insulin-like growth factor-1 (IGF-1) and transforming growth factor-beta (TGF-β) are important cytokines that promote physiological bone turnover (8, 9). IGF-1 is expressed in the fracture callus and facilitates the proliferation and differentiation of osteogenic cells by autocrine function (8, 10, 11). TGF-β also plays a significant role in fracture healing and bone regeneration (10, 11). Furthermore, TGF-β promotes the recruitment of osteoblasts and, eventually, the formation of new bone by stimulating the mobilization of bone marrow mesenchymal stem cells and osteoporotic cells. A decreased expression of TGF-β is seen in smokers (12, 13). The reduction of TGF-β disrupts the migration, proliferation, and differentiation of mesenchymal cells (dependent on the TGF-β signaling pathway) and, in turn, delays ossification and bone repair in smokers (14).

The vitamin D–parathyroid hormone axis (PTH-Vit D) plays an important role in maintaining bone mass density and calcium homeostasis (15). The active form of vitamin D, namely, 1,25 dihydroxycholecalciferol, regulates the absorption of calcium from the intestine (15, 16). Studies have shown that a vitamin D deficiency slows down the healing of broken bones. Smoking reduces bone mass by affecting the absorption of vitamin D and calcium (3, 17, 18). Another hormone that plays an important role in bone regeneration and repair is the parathyroid hormone (PTH) (19). The PTH increases bone mass and prevents osteoporosis and bone fractures (20–23). The stimulation of angiogenesis is one of the mechanisms of bone repair by PTH where, for example, PTH stimulates VEGF secretion in osteoblasts (21, 22). Furthermore, PTH secretion has been shown to be significantly lower in smokers than in non-smokers independently of vitamin D and calcium levels (24).

The available data suggest that WTS use is associated with decreased bone density and osteoporosis (21). Although the role of WTS in cardiovascular, respiratory, and oral diseases has been well-established, there is no report of the effect of WTS on bone healing. Therefore, due to the increasing prevalence of tobacco use in the form of hookah, this study aimed to investigate the effect of hookah smoke on the improving trend of femoral fractures in male rats. In addition, to investigate the possible mechanisms of the effects of WTS on the bone union, several factors affecting bone metabolism, angiogenesis, and repair, including VEGF, PTH, Ca ++, TGF-β, and IGF-1 were measured. Moreover, VEGF and CD34 as angiogenesis indices were assessed in the fracture site by the immunohistochemistry (IHC) method.

## Materials and Methods

### Materials

The study was carried out on 40 Wistar rats, weighing 200–250 g provided by the Kerman University of Medical Science. The animals were kept in a standard environment in the animal house of Afzalipour Medical School, with a controlled temperature (22–24°C) and a 12 h light and 12 h dark cycle with free access to tap water and regular food. Each animal was kept in a separate cage during the study. TGF-β (Cat. No. E-EL-0162), IGF-1 (Cat. No. E-EL-R3001), VEGF (Cat. No. E-EL-R2603), and ELISA assay kits were obtained from Elabscience, Houston, TX, USA. VEGF primary (Cat. No. RC0319) and secondary (MedaView™ Two-step Polymer-HRP Anti-Mouse & Rabbit System, Cat. No. DP0221) antibodies from Medaysis, Livermore, CA, USA, CD34 primary (Cat. No. 503-17544) antibody from Zytomed, Berlin, Germany, and secondary antibody (MedaView™ Two-step Polymer-HRP Anti-Mouse & Rabbit System, Cat. No. DP0221) from Medaysis, USA, were purchased and used in this study. PTH (material No.11972103122), Vit-D (material No. 05894913190), and Ca++ (material No. 05061482190) electrochemical assay kits were obtained from Roche Company, Germany, for Cobas analyzer. Double Apple tobacco was the product of the Al Fakher company of UAE.

### Methods

The experiment was conducted based on the guidelines of the national care and use of laboratory animals (ethics committee permission of the Kerman University of Medical Sciences, Kerman, Iran; IR.KMU.REC.1399.602).

In this study, 40 male Wistar rats were randomly divided into two main groups of 20, including the femoral fracture (Fx) group, which was exposed to room air after a femoral bone fracture, and also the Fx + WTS group, which was exposed to hookah smoke after a femoral bone fracture during the study. The femoral fracture group was divided into two subgroups, Fx28 and Fx42, and the Fx + WTS group was also divided into two subgroups, Fx + WTS28 and Fx + WTS42, which were evaluated for bone healing 28 and 42 days after femoral fracture, respectively.

#### Waterpipe Tobacco Smoking Machine and Exposure to Smoke

**Figure 1:**
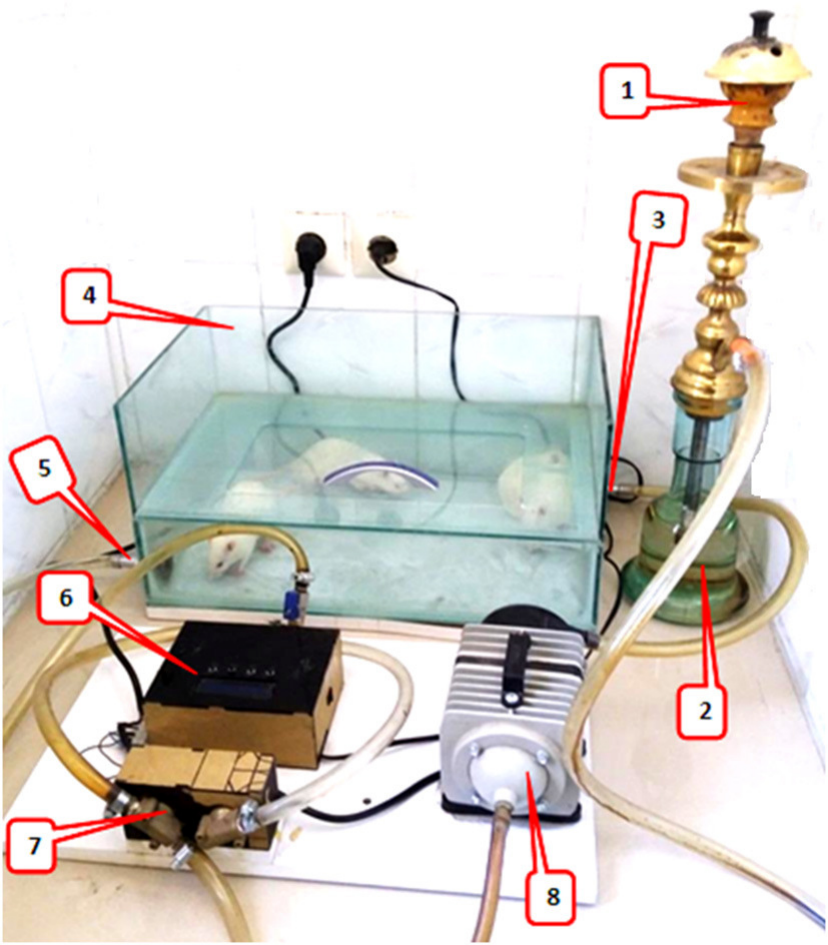
Waterpipe tobacco smoking setup: 1. the ceramic head of hookah for tobacco and charcoal, 2. water jar where the smoke passes, 3. fresh air/waterpipe smoke input, 4. a Plexiglass chamber (8 mm thick), placement for animals to inhale hookah smoke, 5. fresh air/waterpipe smoke output, 6. A timing controller for the sequence of operation of the pump and valve, 7. electronic valve, and 8. A vacuum pump that sucked tobacco smoke from the hookah and, with the help of a regulator, periodically controlled the blowing of smoke and fresh air through the valves into the chamber.

The rats were placed in a plastic container with dimensions of 50 × 30 × 12 cm. The smoke generator had a suction pump that sucked tobacco smoke from the hookah and, with the help of a regulator, periodically controlled the blowing of smoke and fresh air through the valves into and out of the chamber (Figure 1). The tobacco smoke was blown into the chamber for 30 s, then the smoke valve was closed, and the fresh air inlet was opened for 30 s to clear the chamber. Finally, the animals were allowed to inhale fresh air for another 30 s. This cycle was repeated 20 times daily for a total of 30 min per day for 5 days a week for the WTS subgroups during the indicated period (22–24). During smoking, the level of CO concentration was maintained at 907 ± 108 ppm in the chamber using a Testo 310 measurement device (Testo Ltd., Lenzkirch, Germany) (23).

The control subgroups were placed in a smoke-free chamber for 30 min, five times a week to experience environmental protocol stress. The type of tobacco used was Al Fakher Double Apple, which is a well-known brand around the world and is widely consumed in Iran.

#### Surgery, Bone Fracture, and Pinning

**Figure 2:**
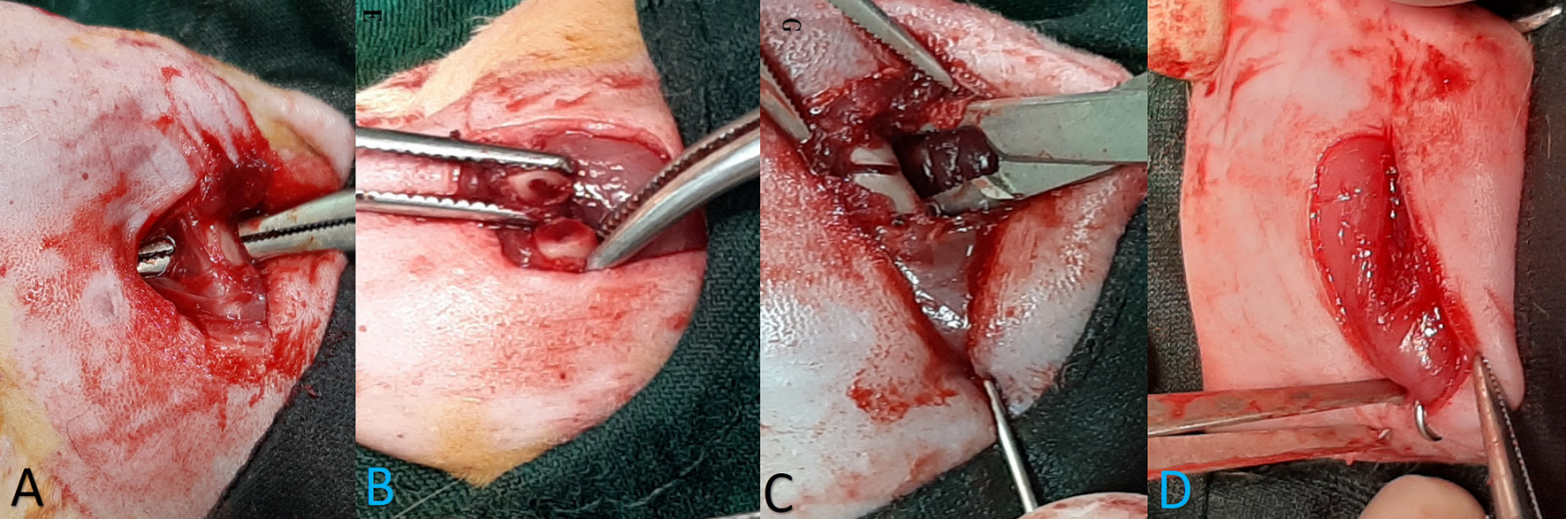
The stages of bone fracture. **(A)** Anesthesia induction was done by the injection of ketamine and xylazine and the maintenance of anesthesia during surgery with 1% isoflurane. **(A)** A lateral incision of the thigh was done and, through intermuscular plan femoral bone, has been exposed. **(B)** Osteotomy site. Osteotomy was performed with an orthopedic saw. Injected saline was used during the incision to prevent heat damage, and the cut was made completely transversely. **(C)** Retrogradely, the pin was inserted in the proximal part and exited from the greater trochanter; then, the reduction was established, and the pin was inserted and continued from the greater trochanter to the distal part of the femur. **(D)** Reduction was controlled visually and by radiography, then a pin was cut and buried under the muscles around the greater trochanter, and wound suturing was performed in anatomical layers.

For surgery and bone fractures, rats were anesthetized with xylazine (10 mg/kg) and ketamine (40 mg/kg). Afterward, 1% isoflurane was inhaled to maintain anesthesia during surgery. After shaving the rat thigh hair and cleaning it with an alcohol swab, a 1.5-cm incision was made in the lateral side of the left thigh. Exposure was performed between the lateral muscles and the bone was exposed. A part of the bone between the greater trochanter and the distal thigh was selected for osteotomy. Attempts were made to avoid injuring the periosteum. Then osteotomy was performed with a thin-blade orthopedic saw in the shaft area. Then, an orthopedic pin with a 1.1-mm diameter was retrogradely passed from the fracture site to the greater trochanter. Thereafter, the reduction was established and the pin was inserted from the greater trochanter and extended distally to the femur. The reduction was checked both visually and by radiography, and if desired, the wound was sutured in anatomical layers. Afterward, the end of the Kirschner (K-) wire was cut and bent in on itself and buried in the muscle area around the greater trochanter (Figure 2). Animals with helical fractures and separate parts were excluded from the study. Immediately after wound healing, one dose of cefazolin (30 mg/kg) was injected into all animals. After surgery, paracetamol (200 mg/kg/daily) was also added to the water bottle of the animals for a week to reduce pain.

#### Radiographic Investigation

Radiographic images were taken from the anteroposterior (AP) and lateral (L) views immediately after surgery and on days 10, 28, and 42 after the fracture. Bone healing was rated using the modified Lane and Sandhu radiological scoring system (25). Two veterinary radiologists scored the radiographic images as double-blinding. The radiographs were rated as 0: without bone formation, 1: <25% bone formation, 2: 25–50% bone formation, 3: 50–75% bone formation, and 4: >75% bone formation. Union scores were as follows, 0: non-union, 1: possible union, and 2: radiographic union. Remodeling scores were as follows, 0: no evidence of remodeling, 2: intramedullary remodeling, and 4: cortical bone remodeling

Finally, the measurements of the three above criteria, namely, bone formation, radiographic union, and cortical bone remodeling, in each sample were done, and the scores were added together for each animal (maximum 10) and finally compared between groups.

#### Histopathological Evaluation of Bone Repair

After the blood sampling, described in section Measurement of Serum Biochemical Factors, the animals were sacrificed for the histological examination of the fracture site under deep anesthesia, and samples were collected from the fracture area and its surroundings. Bone tissue samples with an approximate size of 2 mm were placed in 10% formaldehyde for 24 h and then decalcified in 10% formic acid for 4 days. From the paraffin blocks of the samples, 3–4 micron sections were prepared and stained with H&E. The results of the H&E staining were evaluated and graded by two pathologists who were blind to the groups. Four factors, including soft-tissue edema, granulation tissue precallus formation, and callus formation, were evaluated, and each factor was scored as one plus to four plus. Callus formation indicated a more advanced stage of bone repair and three other factors indicated a more early stage of bone repair, respectively.

#### Immunohistochemistry to Evaluate the VEGF, CD34, and Angiogenesis in the Fracture Site

The vascular endothelial growth factor is a known essential growth factor for vascular endothelial cells and plays a pivotal role in some physiological phenomena, such as angiogenesis and bone formation and healing (26). The hematopoietic progenitor cell antigen CD34 is a transmembrane phosphoglycoprotein that is expressed by CD34-positive endothelial/hematopoietic progenitor cells. They help bone healing by providing an appropriate environment for the fractured bone *via* vasculogenesis/angiogenesis and osteogenesis (27). Immunohistochemistry was performed in order to evaluate the VEGF and CD34 protein markers and angiogenesis. Briefly, 3–4 micron sections were prepared from paraffinic bone tissue samples. To remove paraffin, the lamellae were placed in the oven at 74°C for 15 min. Then dehydration was performed by xylene and 96% alcohol in a ratio of 50:70 distilled water. Afterward, it was placed in a microwave oven at 70°C [for VEGF, we used ethylenediaminetetraacetic acid (EDTA) buffer pH = 8 and for CD34, we used EDTA buffer pH = 6]. The tissues were incubated for 1 h at room temperature with the primary antibody. After washing with Tris-buffered saline (TBS) pH = 7.4, the secondary antibody was added for 15 min at room temperature, and washing with a TBS buffer pH = 7.4 was performed. For the second time, the secondary antibody was added again for 15 min, and washing with TBS buffer pH = 7.4 was performed. To observe the proteins, dye 3, 3′-diaminobenzidine (DAB) (brown) was added. The samples were counterstained with hematoxylin and stained with H&E for 20 s, and then the microvessel properties were evaluated. The positive control for VEGF was the small intestine, and for CD34, it was the lung tissue of the rats. The negative controls were sections treated with the same protocols but with the primary antibody omitted.

#### Measurement of Serum Biochemical Factors

One day after the end of the 4- and 6-week protocols in the different groups, the animals were anesthetized with sodium thiopental (50 mg/kg). Then, 2 cc of blood were taken from the corner of the eye. After clotting, the blood was centrifuged at 3,500 rpm for 15 min, and the serum was separated and stored at −70°C (28). The serum levels of vitamin D, calcium, and PTH were measured by an electrochemical assay. The serum levels of TGF-β, IGF-1, and VEGF were determined using the ELISA method according to the kit instructions. Serum cotinine level, which is a component of tobacco alkaloid and the predominant metabolite of nicotine, was also measured by the ELISA method.

### Statistical Analysis

The sample size was selected based on similar previous studies (29). Additionally, the power analysis for the ANOVA showed that 10 rats per group were required based on a type I error of 5%, power of 0.8. Power calculations were done using the G^*^Power software (Heinrich-Heine-Universität Düsseldorf, Düssel-dorf, Germany; http://www.gpower.hhu.de/) (30). After confirming the normality of parametric data with the Shapiro–Wilk test, a group comparison was performed using a one-way ANOVA and *post-hoc* Tukey tests. Non-parametric data were analyzed by Kruskal–Wallis H and Mann–Whitney U-tests. A value of *p* < 0.05 was considered as the level of significance. Data were presented as mean ± SE. Data analysis was performed using SPSS software version 20 (version 20; SPSS, Inc. Chicago, IL, USA).

## Results

During the study, infection or discharge from the surgical site was not observed in any of the animals in the study groups, and the wound was completely closed in all samples. In addition, no pin exit or displacement was observed in any of the samples.

**Figure 3:**
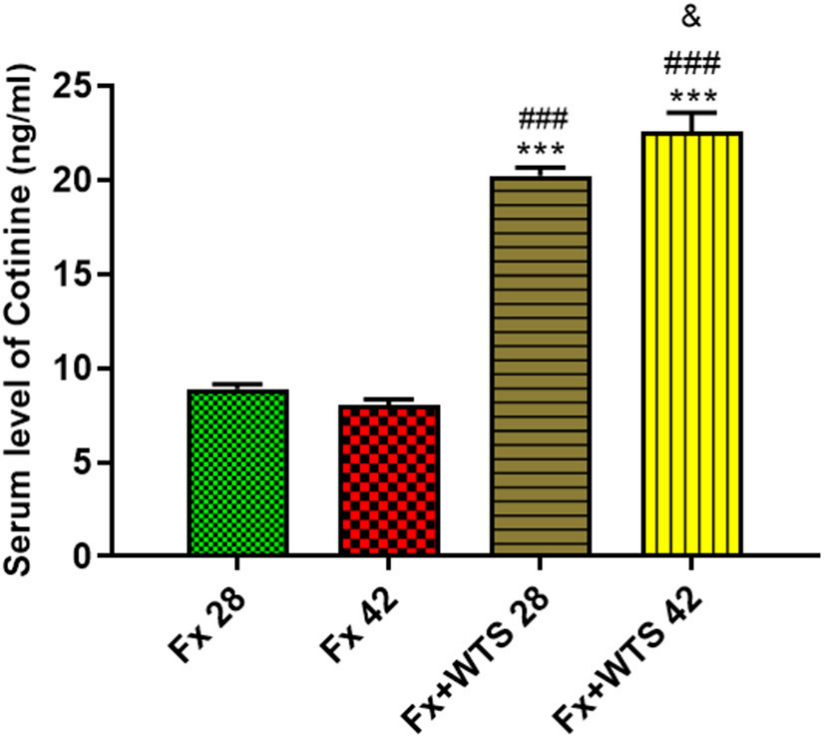
Effects of waterpipe tobacco smoking (WTS) on serum cotinine levels in experimental groups. Fx 28: group subjected to room air for 28 days after surgery and a femoral bone fracture, Fx 42: group subjected to room air for 42 days after surgery and a femoral bone fracture, Fx + WTS 28: group subjected to WTS for 28 days after surgery and a femoral bone fracture, and Fx + WTS 42: group subjected to WTS for 42 days after surgery and a femoral bone fracture. The results are presented as mean ± SEM, *n* = 10. ^***^p < 0.001 vs. Fx 28; ^###^*p* < 0.001 vs. Fx 42; ^&^*p* < 0.05 vs. Fx + WTS 28.

The serum cotinine level in the WTS subgroups were significantly higher than the Fx subgroups (20.24 ± 0.4 ng/ml vs. 8.91 ± 0.24 ng/ml in 28 days and 22.57 ± 0.99 ng/ml vs. 8.08 ± 0.26 ng/ml in 42 days, *p* < 0.001). Additionally, cotinine levels were higher (22.57 ± 0.99 ng/ml vs. 20.24 ± 0.4 ng/ml *p* < 0.05) in the subgroup that received longer smoke for 42 days than in the group that received smoke for 28 days (Figure 3).

### Radiographic Findings

**Figure 4:**
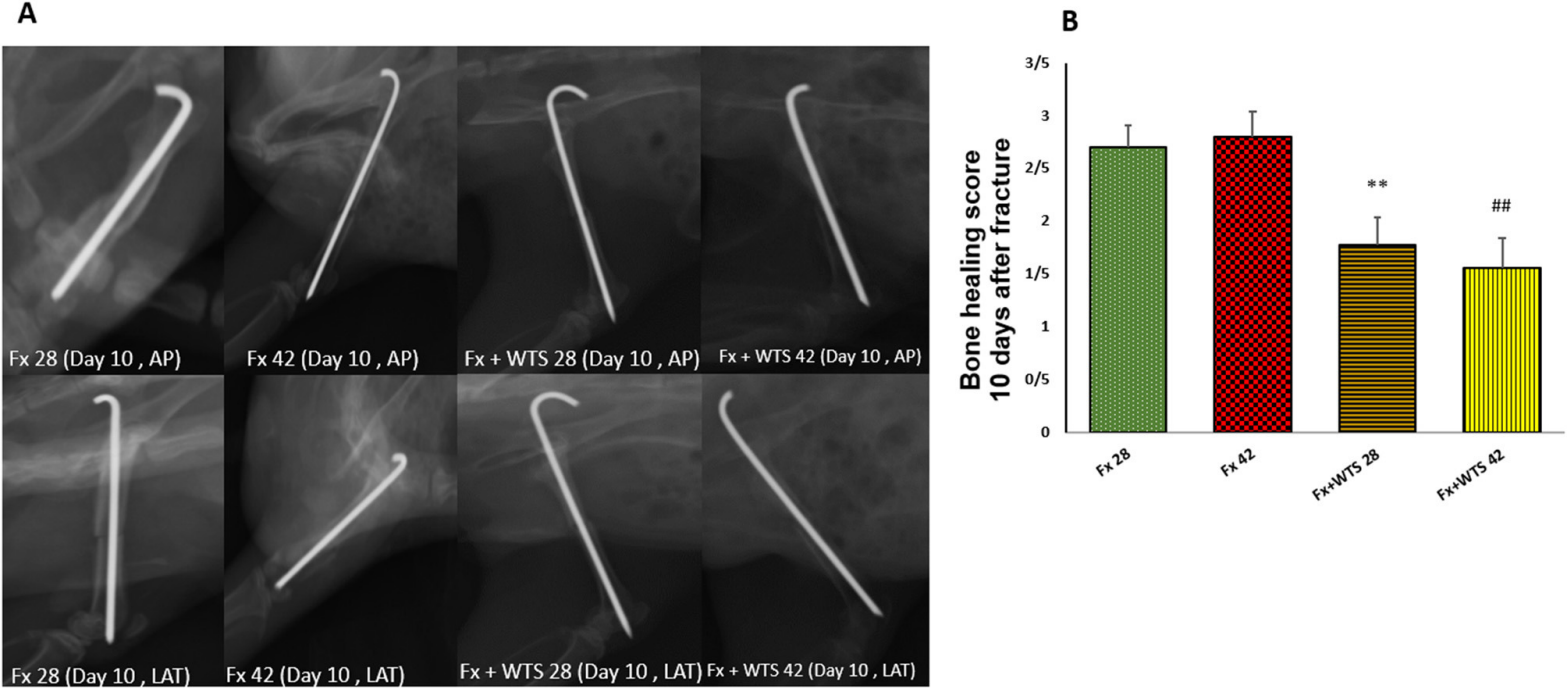
Radiographic evaluation revealed WTS decreased the process of fracture healing 10 days after fracture. **(A)** X-ray images of lateral and AP view of the femur. **(B)** The score of bone healing in experimental groups. Fx 28: group subjected to room air for 28 days after surgery and a femoral bone fracture, Fx 42: group subjected to room air for 42 days after surgery and a femoral bone fracture, Fx + WTS 28: group subjected to WTS for 28 days after surgery and a femoral bone fracture, Fx + WTS 42: group subjected to WTS for 42 days after surgery and a femoral bone fracture. The results are presented as mean ± SE, *n* = 10. ^**^p < 0.01 vs. Fx 28; ^##^*p* < 0.01 vs. Fx 42.

**Figure 5:**
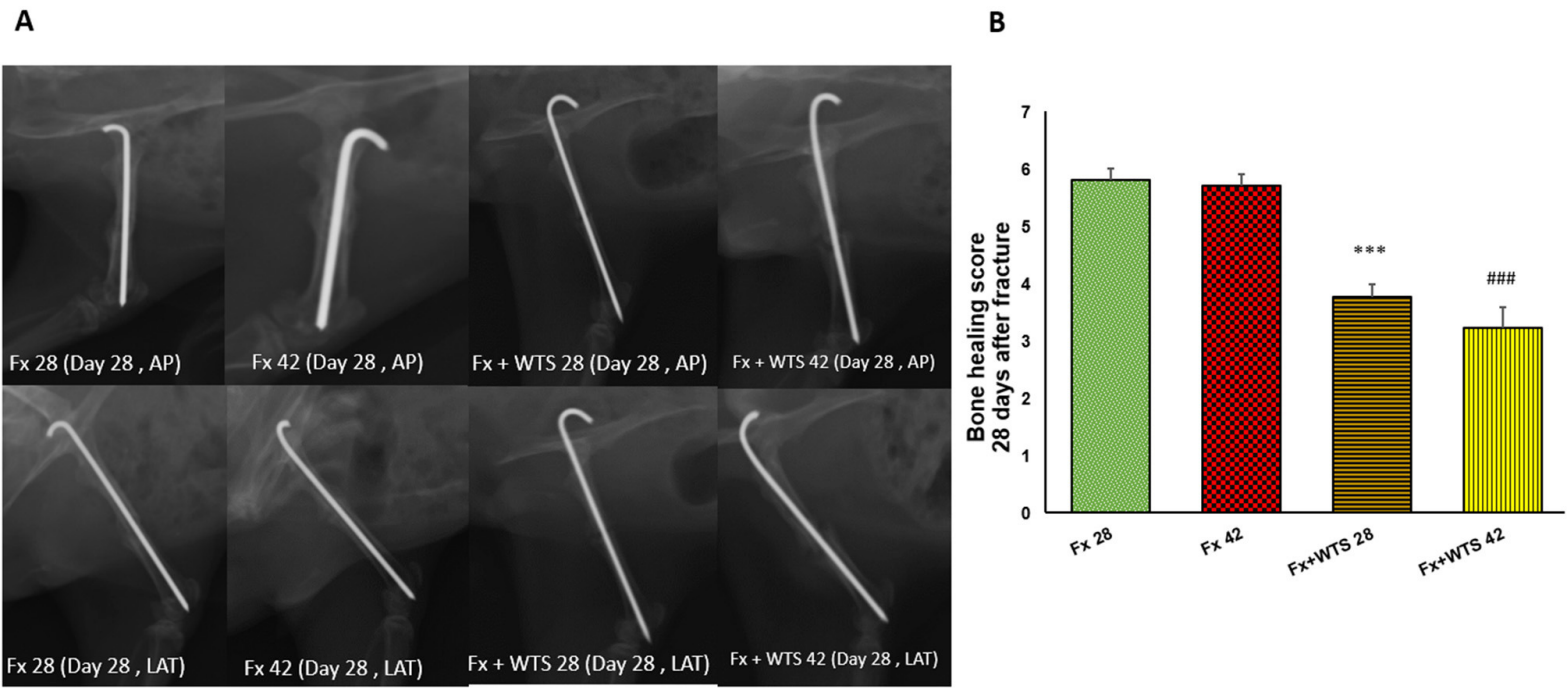
Radiographic evaluation revealed WTS decreased the process of fracture healing in 28 days after fracture. **(A)** X-ray images of lateral and AP view of femur **(B)** the score of bone healing in experimental groups. Fx 28: group subjected to room air for 28 days after surgery and femoral bone fracture, Fx 42: group subjected to room air for 42 days after surgery and femoral bone fracture, Fx + WTS 28: group subjected to Waterpipe tobacco smoking for 28 days after surgery and femoral bone fracture, Fx+WTS 42: group subjected to Waterpipe tobacco smoking for 42 days after surgery and femoral bone fracture. The results are presented as mean ± SE, *n* = 10. ^***^p < 0.001 vs. Fx28; ^###^*p* < 0.001 vs. Fx 42.

**Figure 6:**
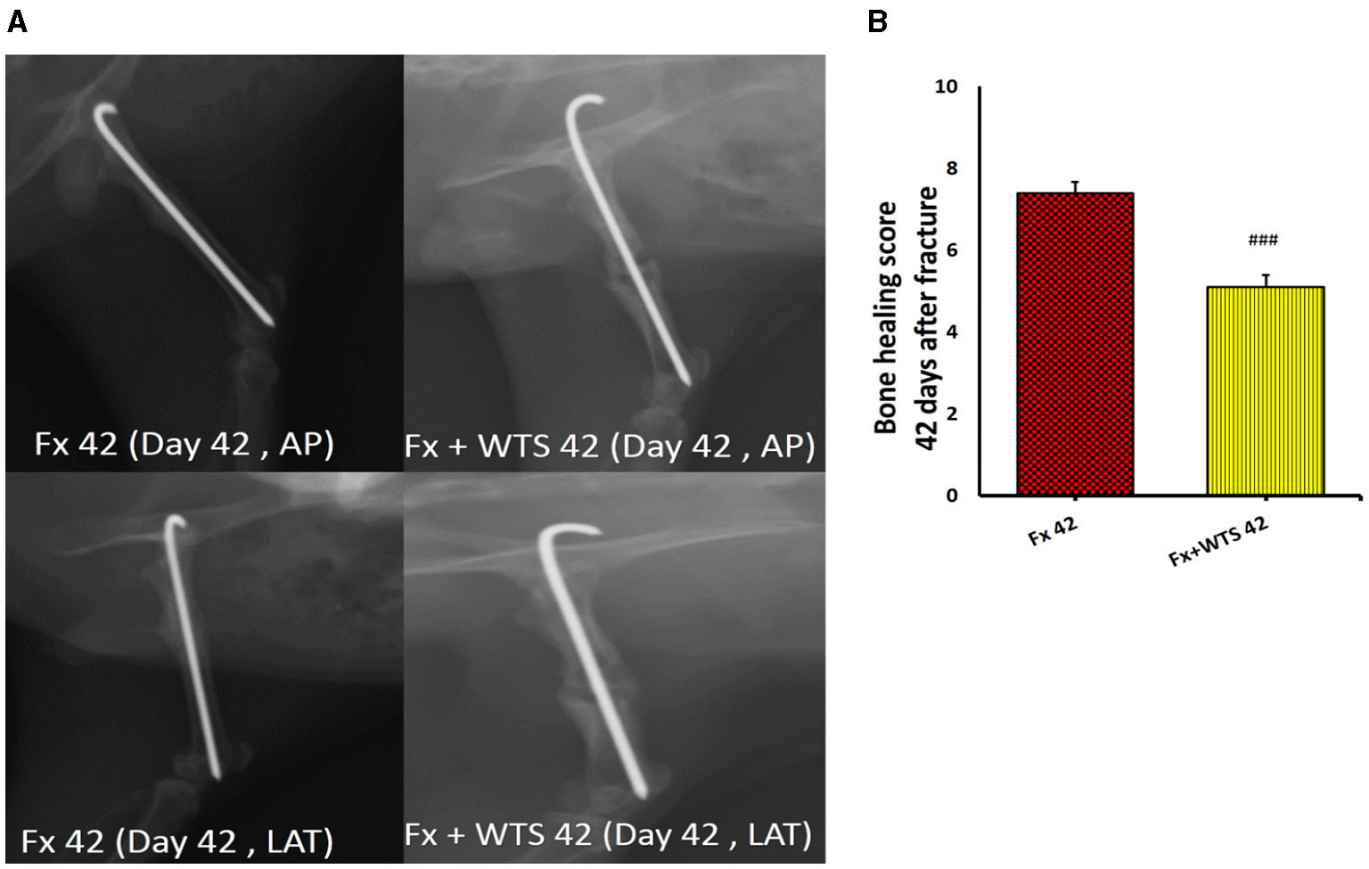
Radiographic evaluation revealed WTS decreased the process of fracture healing 42 days after fracture. **(A)** X-ray images of lateral and AP view of the femur. **(B)** The score of bone healing in experimental groups. Fx 42: group subjected to room air for 42 days after surgery and a femoral bone fracture, Fx + WTS 42: group subjected to WTS for 42 days after surgery and a femoral bone fracture. The results are presented as mean ± SE, *n* = 10. ^###^*p* < 0.01 vs. Fx 42.

A radiographic evaluation revealed that hookah smoke consumption counteracts the progress of the bone repair and union formation and is associated with a decrease in the bone healing score. This negative effect of hookah smoke was observed at 10, 28, and 42 days after fracture. As they have shown, the bone healing scores in Fx +WTS subgroups decreased significantly against the corresponding Fx subgroups after 10 days of smoking (1.77 ± 0.27 vs. 2.7 ± 0.21, *p* < 0.01) (Figure 4) and also after 28 and 42 days of smoking (3.77 ± 0.22 vs. 5.8 ± 0.2 in 28 days and 3.22 ± 0.36 vs. 5.7 ± 0.21 in 42 days, *p* < 0.001) (Figures 5, 6).

### Histopathological and Immunohistochemical Findings

**Figure 7:**
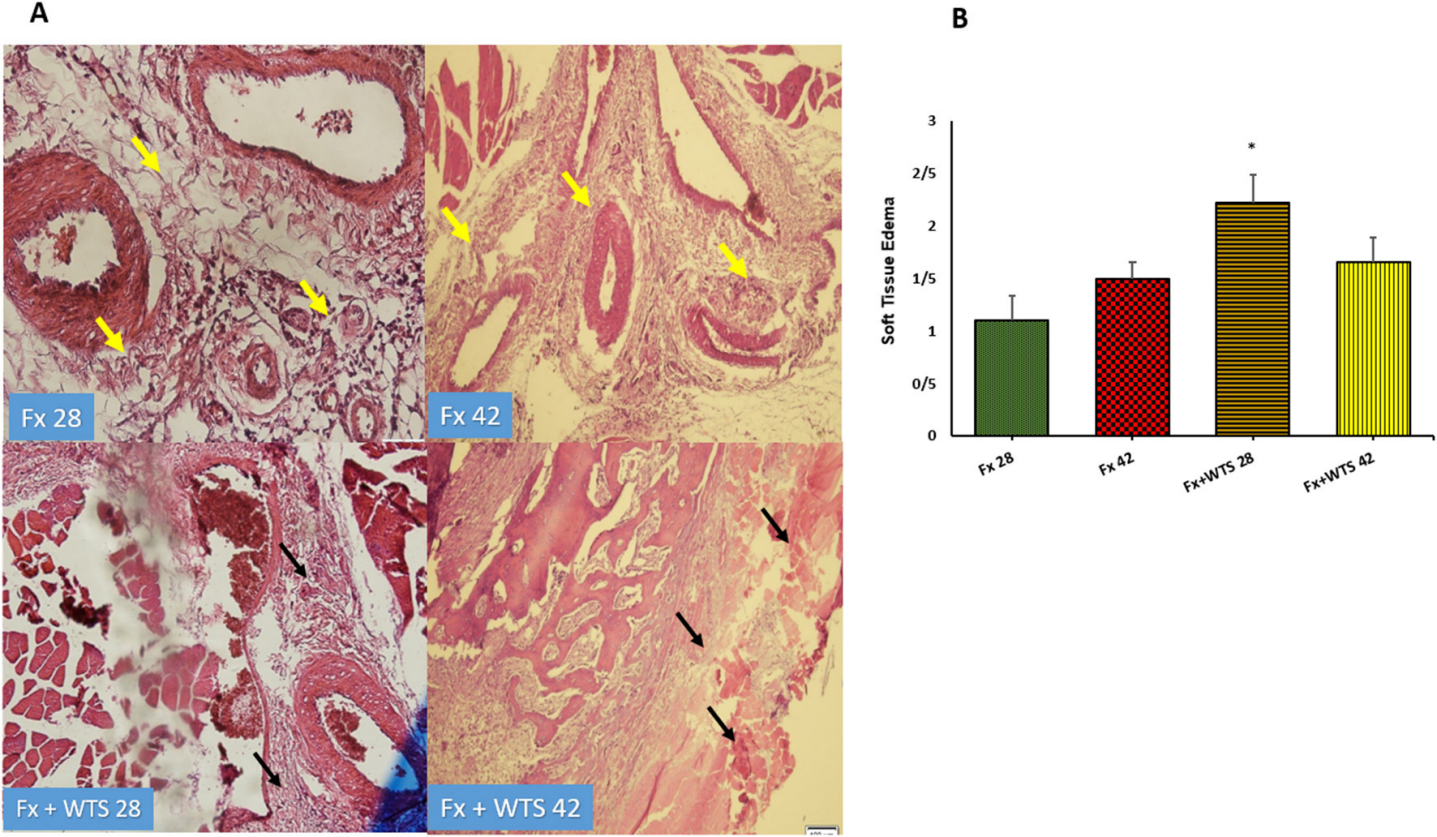
**(A)** Pathological representation of soft tissue edema. In the Fx 28 group, edema has decreased and indicates regular soft tissue without edema (the yellow arrows). In Fx 42 group, the same is true. In Fx + WTS 28 group, the soft tissue around the fracture is completely edematous and irregular and the beginning of the fibrosis process is observed (black arrows). In Fx + WTS 42 group, the surrounding tissue is completely fibrotic and edematous (black arrows). **(B)** The quantification of the soft tissue edema finding in experimental groups. Fx 28: group subjected to room air for 28 days after surgery and a femoral bone fracture, Fx 42: group subjected to room air for 42 days after surgery and a femoral bone fracture, Fx + WTS 28: group subjected to WTS for 28 days after surgery and a femoral bone fracture, Fx + WTS 42: group subjected to WTS for 42 days after surgery and a femoral bone fracture. The results are presented as mean ± SEM, *n* = 10. ^*^p < 0.05 vs. Fx 28.

**Figure 8:**
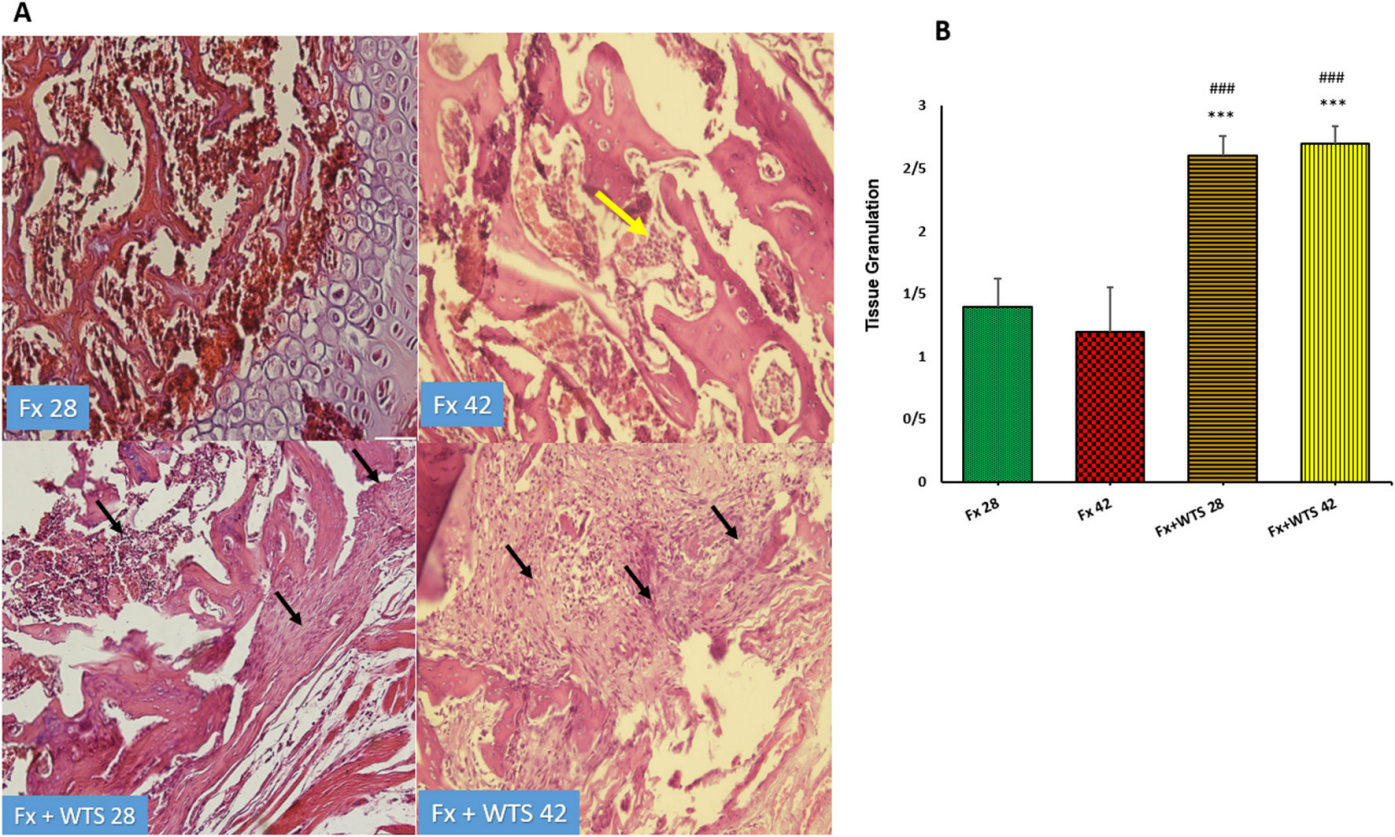
**(A)** Pathological representation of tissue granulation. In the Fx 28 group, tissue granulation is rare. In the Fx 42 group, the yellow arrow indicates that the tissue granulation has been removed and the bone marrow has been replaced. In the Fx + WTS 28 group, a tissue granulation fracture is observed, which is in the early stages of the healing process (black arrow). In the Fx + WTS 42 group, the black arrow indicates the fibrosis of the non-union, and tissue granulation has changed to fibrotic tissue (black arrow). **(B)** The quantification of the pathologic findings regarding tissue granulation in experimental groups. Fx 28: group subjected to room air for 28 days after surgery and a femoral bone fracture, Fx 42: group subjected to room air for 42 days after surgery and a femoral bone fracture, Fx + WTS 28: group subjected to WTS for 28 days after surgery and a femoral bone fracture, Fx + WTS 42: group subjected to WTS for 42 days after surgery and a femoral bone fracture. The results are presented as mean ± SEM, *n* = 10. ^***^p < 0.001 vs. Fx 28; ^###^*p* < 0.001 vs. Fx 42.

**Figure 9:**
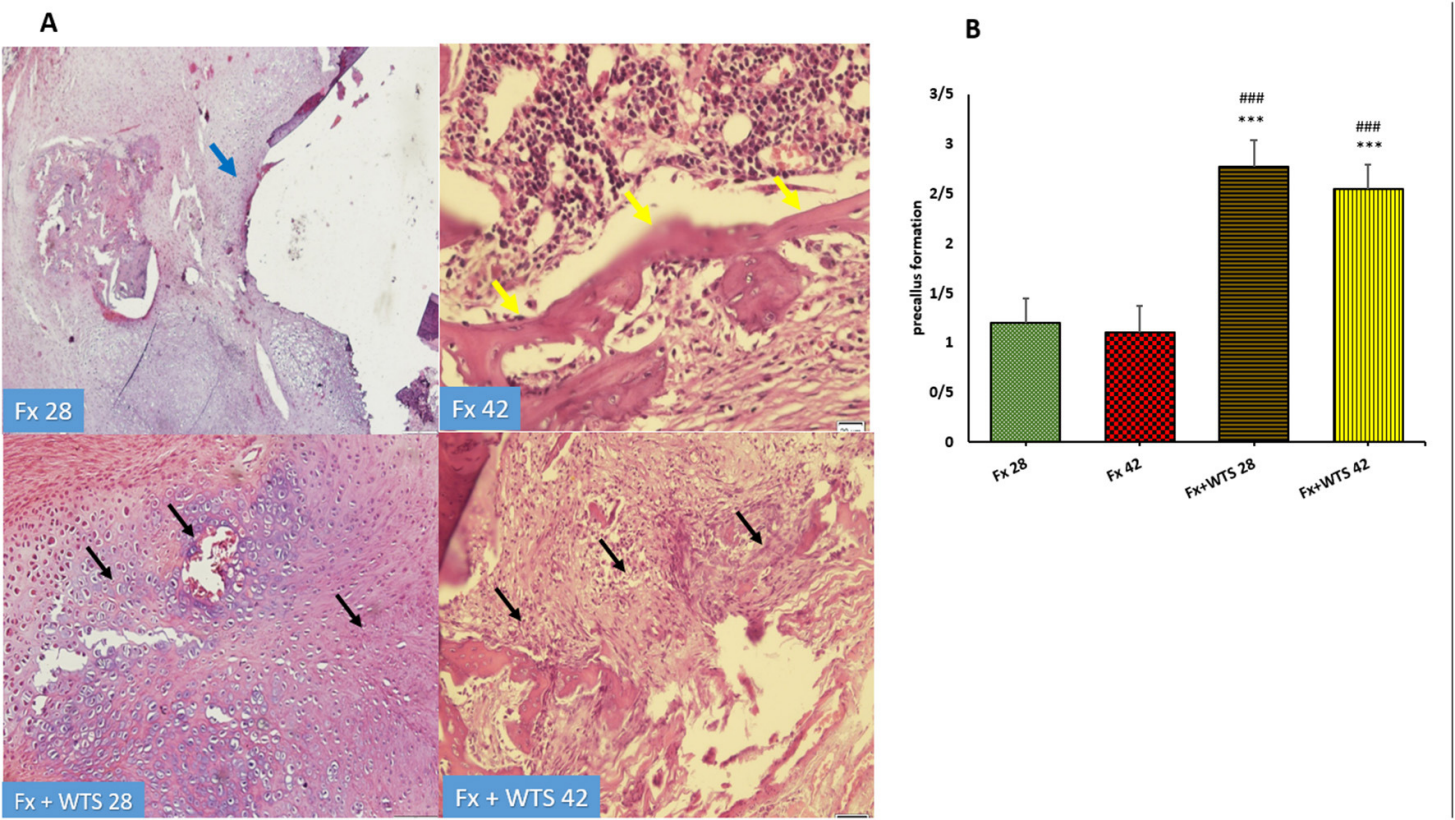
**(A)** Pathological representation of precallus formation. The Fx 28 group does not show precallus tissue, and the conversion of precallus to callus and regular bone is observed (blue arrow). In the Fx 42 group, bone callus formation and a regular osteoblastic rim forming callus can be seen (yellow arrows). The Fx + WTS 28 group indicates the beginning of the healing process and the beginning of bone and precallus tissue repair (black arrow). In the Fx + WTS 42 group, the precallus remnant and fibrotic non-union are observed (black arrow). **(B)** The quantification of pathologic findings regarding precallus formation in experimental groups. Fx 28: group subjected to room air for 28 days after surgery and a femoral bone fracture, Fx 42: group subjected to room air for 42 days after surgery and a femoral bone fracture, Fx + WTS 28: group subjected to WTS for 28 days after surgery and a femoral bone fracture, Fx + WTS 42: group subjected to WTS for 42 days after surgery and a femoral bone fracture. The results are presented as mean ± SEM, *n* = 10. ^***^p < 0.001 vs. Fx 28; ^###^*p* < 0.001 vs. Fx 42.

**Figure 10:**
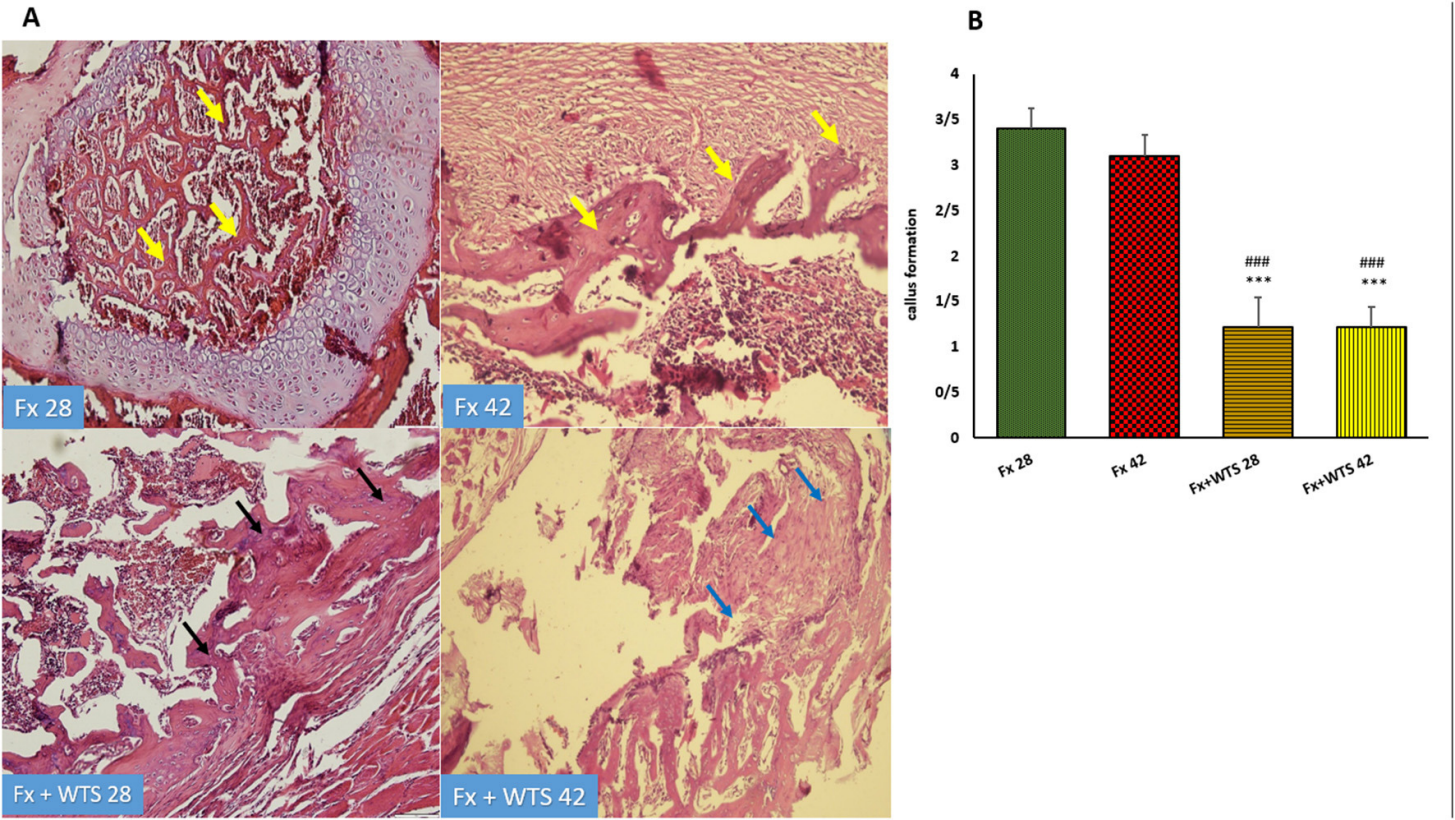
**(A)** Pathological representation of callus formation. In the Fx 28 group, fractures show bone blades that are properly formed and have regular bone marrow between them (yellow arrows). In the Fx 42 group, callus tissue is observed that has formed regularly (yellow arrows). In the Fx + WTS 28 group, pre-callus tissue is still observed, and the callus is irregularly formed and weakly shaped (black arrows). In the Fx + WTS 42 group, fibrotic non-union is observed (blue arrows). **(B)** The quantification of pathologic findings regarding callus formation in experimental groups. Fx 28: group subjected to room air for 28 days after surgery and a femoral bone fracture, Fx 42: group subjected to room air for 42 days after surgery and a femoral bone fracture, Fx + WTS 28: group subjected to WTS for 28 days after surgery and femoral bone fracture, Fx + WTS 42: group subjected to WTS for 42 days after surgery and femoral bone fracture. The results are presented as mean ± SEM, *n* = 10. ^***^p < 0.001 vs. Fx 28; ^###^*p* < 0.001 vs. Fx 42.

**Figure 11:**
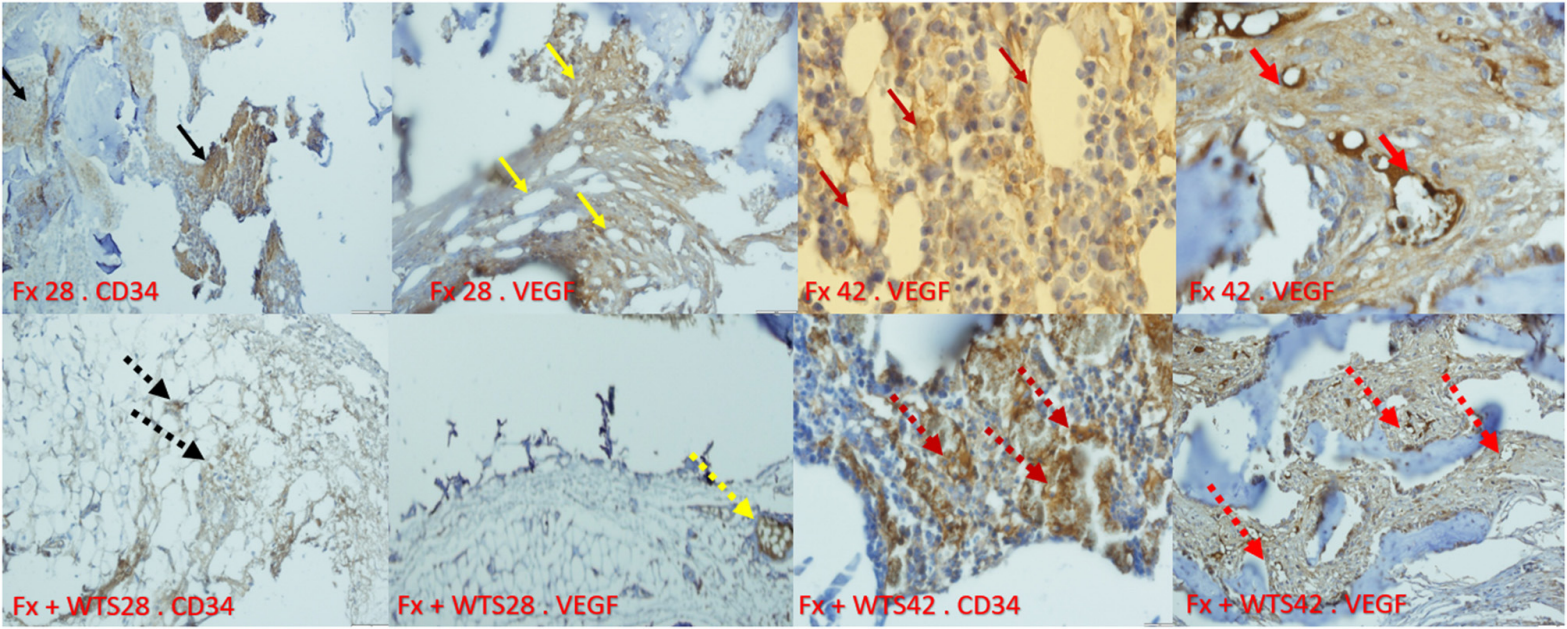
Immunohistochemical representation of the bone fracture area in experimental groups. Fx 28-CD34: The arteries that are properly formed around the fracture tissue (black arrow). Fx 28-vascular endothelial growth factor (VEGF): Represents open arteries and regular vascular tissue and is prominent around the fracture site (yellow arrow). Fx 42-VEGF: Indicates stained osteoblasts and clearly shows VEGF expression in osteoblasts that are active and regular (crimson arrow). Fx 42-VEGF: The vessels inside the callus are actively present and are supplying blood to the repair site (red arrow). Fx + WTS 28-CD34: Small, fragile vessels are shown around the repair site (black arrow point dots). Fx + WTS 28-VEGF: Small vessels and thrombosis are evident at the fracture site (yellow dashed arrow). Fx + WTS 42-CD34: Thrombotic and fragile arteries that are irregular and highly visible at the fracture site (crimson dashed arrow). Fx + WTS 42-VEGF: Small blood vessels inside the fibrotic tissue that are not functioning properly (red dashed arrow). Fx 28: group subjected to room air for 28 days after surgery and a femoral bone fracture, Fx 42: group subjected to room air for 42 days after surgery and a femoral bone fracture, Fx + WTS 28: group subjected to WTS for 28 days after surgery and a femoral bone fracture, Fx + WTS 42: group subjected to WTS for 42 days after surgery and a femoral bone fracture.

The pathological findings indicated the presence of cartilaginous islands in the woven bone, active cartilaginous procallus, and rimming central foci of osteoid formation in the WTS + Fx28 group. At this time, normal endochondral ossification formed in the Fx28 group. In the Fx + WTS28 group, H&E and the IHC for VEGF revealed microthrombi in the lumen of the periosteal and intramedullary of vessels. However, the vessels in the Fx28 group were normal without vasoconstriction and/or microthrombi (Figures 7–11). In the Fx42 group, H&E and the IHC for VEGF and CD34 also showed results similar to Fx28, wherein normal bone, new red marrow formation, and normal vessels in the red marrow were observed. However, the usage of waterpipe smoke for 42 days increased microthrombi in the lumen of the periosteal of vessels. Vessels were collapsed and narrow in the new marrow spaces and intramedullary. In addition, soft tissue bone around fracture-site, cartilaginous procallus islands, periosteal fibrosis, and new woven bone are still present at the fracture sites (Figures 7–11). A histopathological examination indicated that the soft tissue around the fracture site in the Fx +WTS subgroups was edematous and irregular. However, the scoring of soft tissue edema was significant only in the Fx +WTS28 subgroup in comparison with the Fx28 subgroup (2.22 ± 0.27 vs. 1.1 ± 0.23, *p* < 0.05) (Figure 7). Additionally, tissue granulation score was higher in the Fx + WTS subgroups against their corresponding Fx subgroups (2.6 ± 0.16 vs. 1.4 ± 0.22 in 28 days and 2.7 ± 0.14 vs. 1.2 ± 0.16 in 42 days *p* < 0.001) (Figure 8). Procallus and callus formation occurred with a delay in the Fx +WTS subgroups so that the procallus score was significantly higher (2.77 ± 0.27 vs. 1.2 ± 0.24 in 28 days and 2.55 ± 0.24 vs. 1.1 ± 0.27 in 42 days, *p* < 0.001) and callus score was significantly lesser (1.22 ± 0.32 vs. 3.4 ± 0.22 in 28 days and 1.22 ± 0.22 vs. 3.1 ± 0.23 in 42 days, *p* < 0.001) in the Fx +WTS subgroups when compared with their corresponding Fx subgroups (Figures 9, 10).

### Biochemical Factors

**Figure 12:**
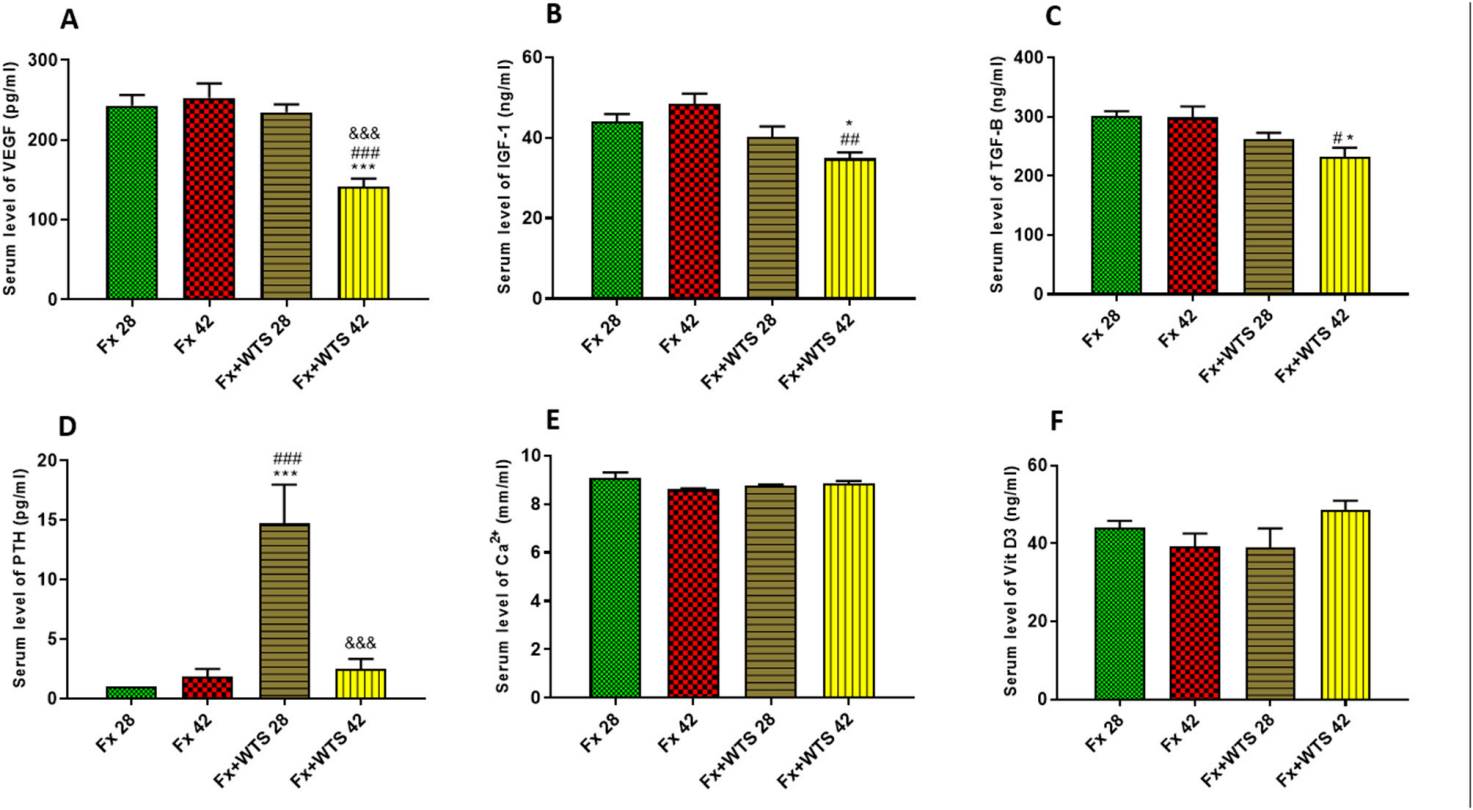
Effects of WTS on the serum levels of **(A)** VEGF, **(B)** insulin-like growth factor 1 (IGF-1), **(C)** transforming growth factor-beta (TGF-β), **(D)** parathyroid hormone (PTH), **(E)** Ca^2+^, and **(F)** vitamin D3 (Vit D3) in experimental groups. Fx 28: group subjected to room air for 28 days after surgery and a femoral bone fracture, Fx 42: group subjected to room air for 42 days after surgery and a femoral bone fracture, Fx + WTS 28: group subjected to WTS for 28 days after surgery and a femoral bone fracture, Fx + WTS 42: group subjected to WTS for 42 days after surgery and a femoral bone fracture. The results are presented as mean ± SEM, *n* = 7. ^*^p < 0.05, ^***^p < 0.001 vs. Fx 28; ^#^*p* < 0.05, ^##^*p* < 0.01, ^###^*p* < 0.001 vs. Fx 42; ^&&&^*p* < 0.001 vs. Fx + WTS 28.

The serum levels of VEGF were significantly lower in the Fx +WTS42 group than other groups (141.25 ± 10.3 pg/ml vs. 252.56 ± 18.22 pg/ml, *p* < 0.001). IGF-1 and TGFβ were significantly lower in the group that received WTS for 42 days compared with control groups (for IGF-1, 36.22 ± 1.8 ng/ml vs. 48.44 ± 2.5 ng/ml, *p* < 0.001 vs. Fx42 and 40.18 ± 2.6 vs. 44.02 ± 1.8, *p* < 0.5 vs. Fx28, respectively) (for TGFβ, 251.32 ± 16.28 pg/ml vs. 289.59 ± 18.6 pg/ml, *p* < 0.05 vs. control groups) (Figure 12). However, serum PTH levels were higher in the Fx + WPS28 group than the Fx28, Fx42, and Fx +WTS42 groups (14.7 ± 3.2 pg/ml vs. 1 ± 0 pg/ml and 1.8 ± 0.65 pg/ml and 2.5 ± 0.84 pg/ml, respectively, *p* < 0.001). The PTH levels reached control levels after 42 days. The serum levels of calcium and vitamin D showed no significant differences in the studied groups.

## Discussion

In this study, the effects of hookah smoke on the healing process and repair of femur fractures were investigated. The radiographic and pathological findings of this study showed that WTS disrupts and delays the healing process of bone fractures. Callus formation, which indicates the more advanced stages of the fracture healing process, was lower in animals exposed to hookah smoke, while the indicators of the more early stages of the union process, including soft tissue edema, granulation tissue, and precallus formation, were significantly higher in these groups. In the presence of WTS, decreasing the amount of some factors, such as VEGF, TGF-β, and IGF-1, that are involved in the healing process of bone fractures and also reducing the amount of angiogenesis is probably the reason for the reduction and delay in bone healing.

Cotinine is the most important metabolite of nicotine. Approximately 70–80% of nicotine in the liver is converted to cotinine (31). Higher serum cotinine levels in rats consuming hookah smoke confirm the effectiveness of the smoking method in this study.

Smoking is considered a risk factor in increasing susceptibility to bone fracture and the length of fracture recovery and delaying bone healing (4). Smoking decreases the bone mineral density, increases the risk of lumbar disc destruction and pelvic fractures, and also enhances the time for bone and wound healing (32). A systematic review with a meta-analysis reported that smokers have double the risk of non-union and also need 27.7 days longer for a union to occur for fractures, osteotomy, arthrodesis, and an established non-union (33). The study of Moghaddam et al. showed that tibial fracture healing was 3–18 times longer in patients who had a history of smoking before and during the fracture than in patients who had no history of smoking (28). Furthermore, animal studies confirmed that exposure to cigarette smoke changes bone matrix composition and worsens bone mineralization (12, 34). Consistent with most studies on cigarettes, our study showed that WTS, even for a short period of 4 weeks, has detrimental effects on bone repair.

The histopathological findings indicated complete bone repair following fracture in control animals after 28 days and/or 42 days as the first hematoma, granulation tissue formation of angioplasia and fibroplasia and inflammatory cells infiltrate, cartilaginous procallus formation, decreasing of angioplasia following endochondral ossification and woven bone formation and finalizing by marrow formation or remodeling of cement line of bony spicules occurred during the repair process. However, in waterpipe smoke-exposed rats, a delay in the bone union at fracture sites was observed. Since the presence of active cartilaginous procallus was found, haphazard new bone formation, fibrotic scar instead of woven bone, decreased angioplasia with endothelial cell hyperplasia and its ballooning, thickened smooth muscle of media, and microthrombi formation in the lumen of vessels in bone union site and soft tissue around the fracture site were detected in the fracture area. These findings were not unexpected because one serving of hookah produces more smoke, CO, and toxins than cigarettes, which enters the lungs of consumers (2) so it can have more destructive effects. Blood vessel regeneration is a vital factor in bone regeneration in the repair phase. In the early stages of broken bone repair, reduced oxygen levels force a large number of chondrocytes to differentiate, causing cartilage callus formation. Mature chondrocytes begin to secrete cartilage matrix factors, and blood vessels grow within the cartilaginous matrix, which increases oxygen levels, thereby leading to osteoblast differentiation and the gradual mineralization of the matrix, followed by an increase in woven bone. Therefore, angiogenesis-related factors are critical for bone union, and any reduction or delay in this pathway will interfere with bone healing (35). VEGF is one of the most important factors related to angiogenesis in the skeletal system (36). Furthermore, VEGF is known as the critical link between angiogenesis and osteogenesis. Several studies have shown that increased VEGF secretion in osteoblasts can increase endothelial cell activity and stimulate angiogenesis (37). Decreased serum VEGF levels in the WTS subgroups, which were exposed to hookah smoke for 42 days, suggested that one of the reasons for the reduced repair and delay in ossification is a decrease in VEGF levels. In addition, the IHC findings showed that the occurrence of angiogenesis in the tissue around the fracture and the callus tissue occurred more regularly in the control groups, while the process of blood supply to the site of injury occurred completely. The regularity and strength of the vessel walls and endothelial cells and the lack of microthrombosis in arterioles and vascular collapse were evident in the histological and immunohistochemical sections of the animals in the control subgroups. In contrast, in the subgroups receiving hookah smoke, the arteries formed were irregular, and the blood supply to the injury site was evidently impaired. The evidence of this was the vascular thrombosis, collapse, and obstruction of the blood supply path in the histological sections and IHC of the smoke-exposed animals. Moreover, 42 days of hookah use reduced the VEGF expression in the fracture sites. The local reduction of VEGF suggested that hookah smoke, in addition to reducing the amount of angiogenesis, may also affect osteoblast differentiation, as previous evidence suggested that VEGF has a direct effect on osteogenic progenitor cells and causes them to differentiate and recruit; hence, stimulating bone repair (38).

Another effective factor in bone growth, strength, and repair is IGF-1. IGF-1 in the bone matrix is supplied by circulating IGF-1 and its local secretion (39). Its amount is directly related to bone mineral density (40). The findings of this study showed that the reduction and delay of bone fracture healing in the smoke-exposed animals for 42 days was significantly associated with a decrease in serum IGF-1 levels. Consistent with the findings of the present study, a recent study by Barbosa et al. indicated that the values of VEGF, IGF-1, and TGF-β decreased in mice exposed to cigarette smoking with tibial fractures (12). It has been reported that, after a fracture, the serum IGF-1 levels in smokers decreased, and this decrease in serum IGF-1 levels in patients was associated with the decreased bone union (41). IGF-1 through its receptor (IGF-1R) activates intracellular kinases such as phosphatidylinositol-3-kinase (PI3K). PI3K activates phosphoinositide-dependent kinase-1 (PDK1, a Ser/Thr kinase), which can phosphorylate and activate protein kinase B (AKT). AKT, in turn, induces osteoblast differentiation by two transcription factors, Runx2 and osterix (42, 43). The deletion of IGF-1R in early osteoprogenitor cells attenuated osteoblast differentiation and proliferation, resulting in bone mass reduction and mineral deposition rates (44).

*In vitro* and *in vivo* studies showed that the accumulated doses of nicotine significantly suppressed chondrocytes and chondrogenic markers (Sox, type II collagen, and aggrecan); hence, delaying chondrogenesis and inducing osteoarthritis. It has been suggested that nicotine exposure suppresses chondrogenesis through the downregulation of the IGF-1/AKT/insulin receptor substrate 1 (IRS-1) signaling pathway (45, 46).

Transforming growth factor-beta is highly secreted by osteoblasts in bone tissue and plays a vital role in controlling bone resorption and ossification (47). The topical administration of TGF-β has been shown to increase and accelerate the healing process of bone fractures (48).

The study of Moghaddam et al. showed that serum TGF-β levels in cigarette smokers with fractures were significantly reduced compared with non-smokers with bone fractures (13). The other animal study demonstrated that nicotine decreases the TGF-β expression and delays bone healing in rabbits (49). Furthermore, the other animal study revealed the reduction of TGF-β levels in mice exposed to cigarette smoke (12). Similarly, the present study showed that the delay and non-union of bone in the Fx + WTS42 subgroup was associated with decreased serum TGF-β levels. TGF-β/bone morphogenic protein (BMP) through both canonical Smad-dependent pathways and the non-canonical Smad-independent signaling pathway (p38 mitogen-activated protein kinase pathway, p38 MAPK) activates Smads and TGF-β activation kinase 1 (TAK1), respectively. These, in turn, trigger the expression of osteogenic transcriptional regulator Runx2 (50).

It seems that long-term waterpipe smoking, partly through the accumulation of nicotine in the body, decreased differentiation and proliferation through the reduction of VEGF, IGF-1, and TGF-β and through vascular constriction, which reduces the bone blood flow and bone capacity to ossification following the fracture. In addition, the CO of smoke has a high affinity to hemoglobin than oxygen (O) and induces a shift of the O dissociation curve to the left. Therefore, it can decrease the oxygen-carrying capacity of blood and oxygen delivery to tissue (51). A reduction in blood-O capacity and the disability of O to dissociate from hemoglobin in the vicinity of tissues, leading to tissue hypoxia and potentially leading further to poor bone healing.

The findings of this study showed that the decrease in IGF-1, VEGF, and TGF- β were not significant in the group exposed to hookah smoke for 28 days compared with the control group. It seems that the changes in the expression of the above factors may depend on the amount and period of hookah smoke and the amount of nicotine accumulation in the body (45). In addition, the amount of cotinine in the 42-day smoke group was higher, which, in turn, can be effective in intensifying the reduction of these factors. On the other hand, the findings of this study showed that PTH was significantly increased in the Fx + WTS28 subgroup. Various studies have shown the beneficial effect of PTH in repairing bone fractures (52). However, our findings showed that, despite the increase in PTH in animals exposed to hookah smoke for a short time, the wound healing and healing process were still delayed. Failure to repair a bone fracture may be due to a delay in release or a large and persistent increase in PTH. Several studies have reported that the intermittent administration of low doses of PTH increases bone synthesis, increases mechanical properties, and stimulates bone repair (16, 21). However, continuous exposure to PTH and overproduction has been shown to cause bone loss (23, 53).

Since previous studies have reported that cigarettes promote osteoporosis and reduce bone mass by reducing the intestinal reabsorption of calcium and vitamin D, we measured these parameters, too (3, 17, 18, 54, 55). In the present study, the serum calcium levels in WTS animals did not change compared with the control group. However, this finding cannot refute or prove the effect of hookah smoke on the absorption of calcium from the intestine. This is because serum calcium is regulated by various hormones such as PTH, vitamin D, and PTH-related protein (PTHrP) (56). Nonetheless, previous studies have shown a wide range of serum calcium levels (3, 57). Although a decrease in vitamin D has been reported in smokers (41), our findings showed that the amount did not change in the WTS group after a fracture. It seems that the main cause of hypovitaminosis D in smokers is attributed to skin aging (58). Despite the importance of the role of vitamin D in bone metabolism, its role in improving acute fractures is less clear (3).

Overall, this study showed that long-term waterpipe smoke inhalation is associated with a decrease in the expression of growth factors, namely, angiogenic and osteogenic markers such as IGF-1, VEGF, CD34, and TGF- β, and the impairment of bone healing. These findings are consistent with the results of studies on bone healing in cigarette smokers, which confirmed that smoking is a risk factor for the development of non-union healing. As discussed above, a part of these adverse effects is attributed to nicotine, which is one of the most important substances in cigarette smoke and hookah (59).

## Conclusion

Based on radiographic, histopathological, immunohistochemical, and biochemical findings, this study showed that hookah smoke consumption delays the healing process and repair of broken bones in male rats. The results of this study can pave the way for clinical studies in this field and ultimately raise awareness about the limitation of hookah use in communities.

## Data Availability Statement

The raw data supporting the conclusions of this article will be made available by the authors, without undue reservation.

## Ethics Statement

The animal study was reviewed and approved by the Ethics Committee of Kerman University of Medical Sciences, Kerman, Iran.

## Author Contributions

SJ devised the main conceptual ideas and along with AS. MShe designed the study. All the authors contributed to the acquisition, the performing of procedures and tests, analyses, or the interpretation of data, the drafting of the manuscript, and approved the final version of the manuscript.

## Funding

The finances of this study were supported by a grant (No: IR.KMU.REC.1399.602) from the Vice-Chancellor for Research and Technology of the Kerman University of Medical Sciences, Kerman, Iran, which is acknowledged. This study was provided from the results of the MD thesis for the orthopedic specialty of MShe.

## Conflict of Interest

The authors declare that the research was conducted in the absence of any commercial or financial relationships that could be construed as a potential conflict of interest.

## Publisher's Note

All claims expressed in this article are solely those of the authors and do not necessarily represent those of their affiliated organizations, or those of the publisher, the editors and the reviewers. Any product that may be evaluated in this article, or claim that may be made by its manufacturer, is not guaranteed or endorsed by the publisher.

## Abbreviations

WTS: waterpipe tobacco smoking
Fx: fracture
VEGF: vascular endothelial growth factor
PTH: parathyroid hormone
TGF-β: transforming growth factor-beta
IGF-1: insulin-like growth factor 1
IHC: immunohistochemistry
CO: carbon monoxide
ppm: parts per million
AP: anteroposterior
L: lateral
H&E: hematoxylin and eosin
EDTA: ethylenediaminetetraacetic acid
ELISA: enzyme-linked immunosorbent assay
ANOVA: analysis of variance
PTHrP: parathyroid hormone-related protein
BMP: bone morphogenetic protein
PDGF: platelet-derived growth factor
PI3K: phosphatidylinositol-3-kinase
AKT: activate protein kinase B
PDK1: phosphoinositide-dependent kinase-1
BMP: bone morphogenic protein
p38 MAPK: p38 mitogen-activated protein kinase pathway
TAK1: TGF-β activation kinase1
IRS-1: insulin receptor substrate 1.
